# Research on a Traffic Sign Recognition Method under Small Sample Conditions

**DOI:** 10.3390/s23115091

**Published:** 2023-05-26

**Authors:** Xiao Zhang, Zhenyu Zhang

**Affiliations:** 1 College of Information Science and Engineering, Xinjiang University, Urumqi 830017, China; 15529319347@163.com; 2 Key Laboratory of Multilingual Information Technology in Xinjiang Uygur Autonomous Region, Xinjiang University, Urumqi 830017, China

**Keywords:** few-shot learning, target detection, FSOD, feature fusion, traffic signs

## Abstract

Traffic signs are updated quickly, and there image acquisition and labeling work requires a lot of manpower and material resources, so it is difficult to provide a large number of training samples for high-precision recognition. Aiming at this problem, a traffic sign recognition method based on FSOD (few-shot object learning) is proposed. This method adjusts the backbone network of the original model and introduces dropout, which improves the detection accuracy and reduces the risk of overfitting. Secondly, an RPN (region proposal network) with improved attention mechanism is proposed to generate more accurate target candidate boxes by selectively enhancing some features. Finally, the FPN (feature pyramid network) is introduced for multi-scale feature extraction, and the feature map with higher semantic information but lower resolution is merged with the feature map with higher resolution but weaker semantic information, which further improves the detection accuracy. Compared with the baseline model, the improved algorithm improves the 5-way 3-shot and 5-way 5-shot tasks by 4.27% and 1.64%, respectively. We apply the model structure to the PASCAL VOC dataset. The results show that this method is superior to some current few-shot object detection algorithms.

## 1. Introduction

A traffic sign intelligent recognition system is the main content of an intelligent transportation system and advanced driving assistance system. It can not only ensure the safe operation of vehicles, but also improve the transportation capacity and make the economy develop rapidly. It has important economic value.

Traffic sign detection and recognition methods based on deep learning has been a research hotspot in recent years. In 2016, Yang Y et al. [1] proposed a traffic sign recognition algorithm, which consists of a region proposal module (RPM) and a classification module (CM). Although the algorithm improves the performance of traffic sign recognition, there are still some problems due to its large resource consumption. In 2018, Jia L and Zengfu W [2] used a fast convolutional neural network (CNN) and a MobileNet structure to refine the positioning of small traffic signs and improve detection performance, but training and detection take a long time. In 2019, Zhu et al. [3] proposed a convolutional network named qNet and sqNet. This network uses a unified macro structure and deep separable convolution for fast traffic sign recognition, which has higher efficiency in parameters and calculations. In the same year, Liu Z et al. [4] proposed a traffic sign detection and recognition method that integrates multi-scale regions, which has good performance in small traffic sign detection. Yuan et al. [5] proposed an end-to-end learning method. For small-scale targets, this method can learn more effective features, but it consumes a lot of resources. In 2020, Chung J H et al. [6] proposed an attention-based convolutional pooling neural network (ACPNN), which combines the attention mechanism to obtain key features, and replaces the maximum pooling with convolution pooling to improve the recognition accuracy of the model in harsh environments. In the same year, Liu Z et al. [7] proposed a detection method called DR-CNN, which is based on deconvolution region and performs well in small target traffic sign detection and recognition. In 2021, Liu Z et al. [8] proposed a traffic sign detection and recognition network called SADANet. This method uses the mapping relationship between image representation and multi-scale features to effectively align the domain distribution of different scales. In the same year, Shen et al. [9] proposed an effective multi-scale attention module, which aggregates features of different scales, suppresses clutter information in the background, and constructs an information feature pyramid for detecting traffic signs of different sizes. In 2022, Dubey et al. [10] proposed a two-step TSR method, which uses a convolutional neural network as a multi-class classifier. Compared with other similar methods, this method has better classification accuracy.

Although some classical object detection algorithms have been applied in practice, most object detection algorithms rely on large-scale labeled data sets to ensure accuracy. However, in real life, it is difficult to obtain labeled data in some fields, or the cost of acquisition is very high. For example, with the continuous development of science and technology, traffic signs are updated rapidly, and the collection and labeling of data sets will consume a lot of manpower, material, and financial resources.

In response to this problem, more and more researchers have begun to combine the few-shot learning method [11] with the target detection framework to construct a few-shot objection detection technology for target detection in the case of few samples [12,13,14]. Small sample target detection is more based on the two-stage target detection method. The detection model is optimized by using the base class and the new class sample set to realize the detection of image targets in the new class. For example, references [15,16,17] used Faster R-CNN as the basic model. In this paper, the two-stage detection method is used and improved on this basis.

Small sample image target detection should not only pay attention to the high-level semantic information of the classification task, but also extract the low-level image semantic information and give the exact position of the target in the image. However, since the support set can provide too few training samples, it is prone to overfitting phenomenon. Secondly, the target category to be detected has only a small number of labeled samples for training. When detecting new categories, many candidate boxes unrelated to the target object are generated, which affects the detection accuracy of the target category. In addition, due to the large scale difference between the support set and the query set images, it also brings some difficulties to task detection and recognition.

In order to solve these problems, we propose a traffic sign detection and recognition method based on FSOD. The main contributions of this paper are as follows:

1. The basic model structure is adjusted so that the network can pay more attention to the detail information and semantic information of the image, reduce the loss of image features, reduce the risk of overfitting, and improve the detection performance in the case of few samples.
2. The ACBAM-RPN module combined with a multi-attention mechanism is proposed to make the model focus on the candidate boxes related to the target category, reduce the number of irrelevant candidate boxes, and improve the detection accuracy of the target category.
3. The multi-scale FPN module is introduced to extract features, and the deep cross-correlation between support set image features and query set image features is calculated to obtain more accurate candidate boxes.

## 2. Methodology

### 2.1. Background of FSOD

FSOD [17] is a target detection and recognition method. Traditional object detection and recognition algorithms require a large amount of labeled data to train the model to accurately detect and classify objects in images. However, collecting such a large data set is usually expensive and time-consuming. FSOD aims to learn new categories of object detection capabilities through a small amount of labeled data. Its core idea is to learn the general representation of object detection tasks by training on a small amount of labeled data, and then apply this general representation to new object detection tasks; that is, use a small amount of labeled data for rapid training and adaptation to new tasks.

**Figure 1:**
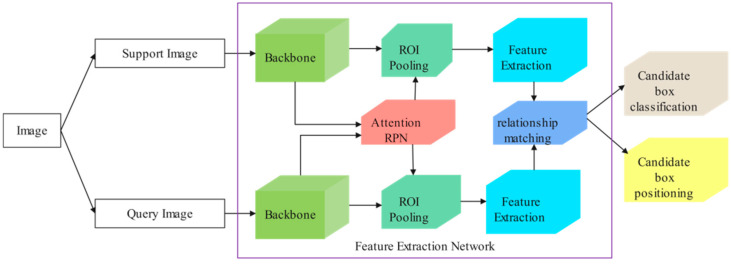
Network structure diagram of FSOD. The overall network structure consists of two parts: Support set branch and query set branch.

The FSOD model consists of two branches: support set and query set. The support set usually contains a small amount of annotation data and corresponding annotation boxes, while the query set contains images of new categories that the network has not seen. The task of the detector is called N-way K-shot detection, where the support set contains a total of N categories and each category has K samples. The design of this detector can improve the efficiency and accuracy of the model in small sample detection tasks. The FSOD model is shown in Figure 1.

### 2.2. Improved Network Structure

**Figure 2:**
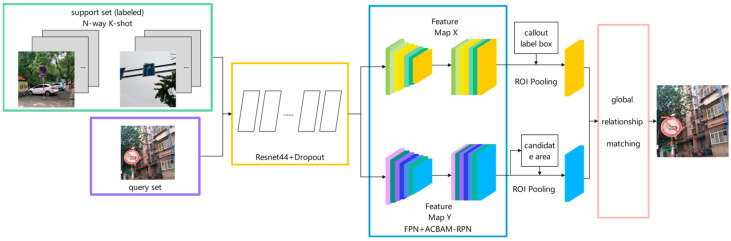
Network structure diagram of improved FSOD. The overall network structure consists of two parts: support set branch and query set branch.

This paper demonstrates the optimization based on the FSOD network, and the improved structure is shown in Figure 2.

In this paper, FPN network and RPN network with an attention mechanism are introduced into query image branch and support set image branch. Specifically, in the support set image branch, the support set image features are input into the FPN network, and each support image feature pyramid is obtained through the channel attention mechanism, which enriches the support set scale space. After obtaining the support image feature pyramid, the multi-scale prototype vector of each class is generated by the weighted prototype network. The second is the query image branch. The query image is first extracted by the FPN network to obtain the feature pyramid, and then input to the attention RPN module designed in this paper to generate candidate regions. Another input of the RPN module is the extracted support set image feature pyramid. In the attention RPN network, the query set feature map of each scale and the support set feature map of the same scale generate the attention feature map, then generate the RoI feature map through the RPN network, and finally generate the corresponding RoI feature vector.

## 3. Materials and Methods

### 3.1. Adjustment of Backbone Network

**Figure 3:**
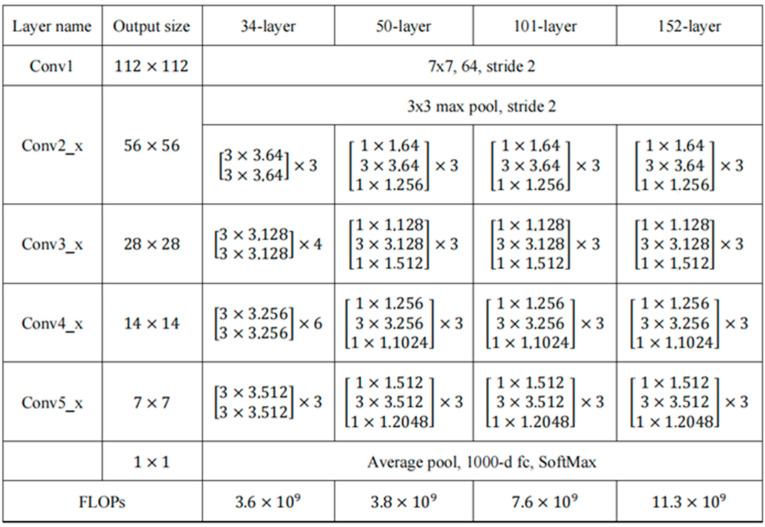
Resnet series backbone network structure.

FSOD uses Resnet50 model [18] as the backbone network, and the network structure of Resnet series is shown in Figure 3. However, during the preliminary experiments, it was found that there was a serious overfitting problem, which was manifested as high accuracy on the training set but low accuracy on the test set. To solve this problem, the network structure of Resnet50 is adjusted in this part.

Specifically, the convolution layers of the first two stages and the last stage of the network are kept unchanged to ensure the basic feature extraction ability of the network. At the same time, the number of shallow network layers is increased; that is, two residual modules are added in the third stage to improve the ability to extract image details, thereby improving the recognition accuracy. In addition, the number of middle layer network layers is reduced; that is, four residual modules are deleted in stage 4 to reduce network computation and detection time. Through these adjustments, the total number of convolution layers of the network is reduced from 50 layers to 44 layers. Such adjustments make the network pay more attention to the detail information and semantic information of the image, reduce the loss of image features so as to better extract the traffic sign image features, and improve the detection performance in the case of few samples.

Finally, this section adds dropout to the fully connected layer of the network, randomly sets some features of the input data to 0, and discards a certain proportion of features, so that the data seen by the network is different during each training, thereby reducing the complexity of the model and the risk of overfitting. The improved Resnet network structure is shown in Figure 3.

Through these adjustments, we better solve the problem of Resnet50 model overfitting. The accuracy on the query set has also been significantly improved. This improvement lays the foundation for the subsequent design and optimization of the model.

### 3.2. RPN Module with Multi-Attention Mechanism

In the traffic sign detection and recognition experiment of FSOD, we found that the data set to be detected contains fewer training samples, and the region proposal network used for model training needs to be trained on a large number of base classes, which will cause the network to generate many candidate boxes unrelated to the traffic signs to be detected and identified, thus reducing the accuracy of detection. To solve this problem, this section proposes a RPN network based on a fusion multi-attention mechanism.

**Figure 4:**
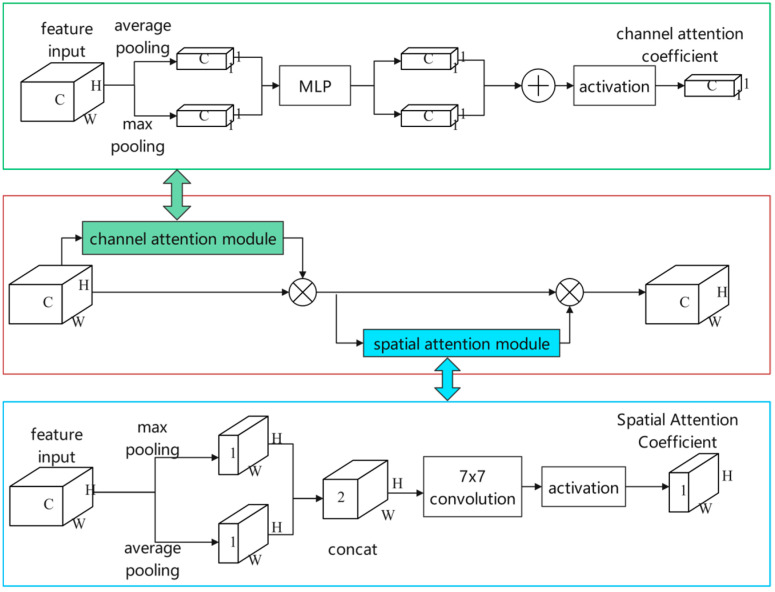
CBAM model structure diagram.

CBAM [19] (Convolutional Block Attention Module) is an attention module for image classification tasks. The CBAM module consists of two parts: Channel Attention Module (CAM) and Spatial Attention Module (SAM). The main idea of the module is to improve the expression ability of the model by learning channels and spatial attention weights on the feature map. The channel attention weight is used to adaptively weigh the features of different channels to improve the channel perception ability of the model. The spatial attention weight is used to adaptively weigh different spatial positions on the feature map to improve the spatial perception ability of the model. The structure of CBAM module is shown in Figure 4.

The channel attention module is used to enhance the expression ability of CNN on the channel dimension. Generally, the output of the convolutional layer consists of multiple channels, each channel corresponding to a feature map. The channel attention module adaptively adjusts the weight of the feature maps of different channels by learning a channel weight vector to enhance the expression of important features and suppress the interference of unimportant features. This channel weight vector is calculated by a global average pooling and two fully connected layers. For the feature map with input size of H×W×C, the CBAM module performs global average pooling and maximum pooling respectively to obtain two compressed feature maps. Then, these two feature maps are input into the multi-layer perceptron (MLP) for dimension reduction and dimension increase, and the weight vector used to represent the importance of the channel is extracted. The weight vector is used to calculate the channel attention weighting coefficient, which can be applied to each channel in the input feature map to emphasize the most important channel for a specific task.

The calculation formula of this sub-module is shown in Formulas (1) and (2). Among them, *FC_max*(*A*) and *FC_avg*(*A*) represent the feature vectors of input feature map A under maximum pooling and average pooling, respectively. *W*_0_ and *W*_1_ refer to the two layers of weights in MLP. The σ refers to the Sigmoid activation function, which is used to scale the output value between 0 and 1. The final channel attention weight coefficient will be used to weight each channel in the input feature map A to generate a weighted output feature map.

(1) $$N_{C}\left(A\right)=\sigma \left(\left[MaxPool\left(A\right);AvgPool(A)\right]\right)$$

(2) $$N_{C}\left(A\right)=\sigma \left(W_{1}\left(W_{0}\left[\left[A_{max}^{C};A_{avg}^{C}\right]\right]\right)\right)$$

During neural network training, the channel attention module may pay too much attention to some feature channels and ignore other important feature channels, thus affecting the robustness of the model. In order to solve this problem, this part improves the original CBAM channel attention module, splices and fuses the two groups of features after pooling, and then trains and optimizes the weight parameters *W*_0_ and *W*_1_ through the multi-layer perceptron, thereby improving performance of the model. MLP is composed of two FC layers. The input features of the first FC layer are reduced in dimension to obtain features, and the hidden layer is activated to obtain the output features. Although the dimension of the weight vector that represents the importance of the channel extracted before and after the improvement is the same, the first FC layer that is trained after feature fusion has more weight parameters. In addition, the second FC layer can mix and calculate the two parts of features, so as to better fit the correlation between channels, which is beneficial to calculate the mutual information of the two sets of features and enhance the expression of key channel features. The process is shown in Formulas (3) and (4), where [*FC_max*(*A*); *FC_avg*(*A*)] is the fusion feature after stitching.

(3) $$N_{C}\left(A\right)=\sigma \left(\left[FC\_max\left(A\right);FC\_avg(A)\right]\right)$$

(4) $$N_{C}\left(A\right)=\sigma \left(W_{1}\left(W_{0}\left[\left[A_{max}^{C};A_{avg}^{C}\right]\right]\right)\right)$$

**Figure 5:**
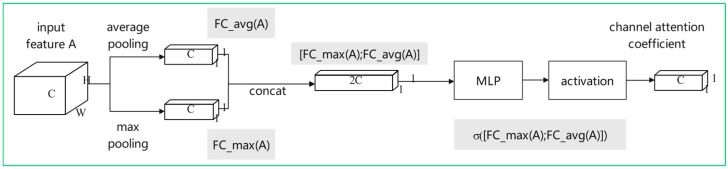
Improved channel attention module structure diagram.

Figure 5 shows the improved structure.

The spatial attention module is used to enhance the expression ability of CNN in the spatial dimension. It adaptively adjusts the contribution of feature maps at different locations by learning a spatial weight vector to enhance the expression of important locations and suppress the interference of unimportant locations. Firstly, the input feature A’ is processed, and the global maximum pooling and average pooling compression channels are used to transform the multi-channel features into single channels. Then, two single-channel feature maps are spliced and compressed at the spatial level using a 7 × 7 convolutional layer. Finally, the weighting coefficient *N_S_* (*A*′) is obtained by using the Sigmoid activation function, as shown in Formulas (5) and (6), where f represents the convolution operation, 7 × 7 is the size of the convolution kernel, and the meaning of other symbols is the same as above.

(5) $$N_{S}\left(A^{\prime }\right)=\sigma \left(f^{7\times 7}\left[FC\_max\left(A^{\prime }\right);FC\_avg\left(A^{\prime }\right)\right]\right)$$

(6) $$N_{S}\left(A^{\prime }\right)=\sigma \left(f^{7\times 7}\left[{A^{\prime }}_{avg}^{S};{A^{\prime }}_{avg}^{S}\right]\right)$$

Based on the above description, this paper designs the ACBAM module to multiply the output of the improved channel attention module and the output of the spatial attention module to obtain the final attention weight, which is then applied to the original feature map to obtain the enhanced feature map, thereby improving the performance of CNN.

### 3.3. Multi-Scale Feature Fusion Module

When the training samples cover a large number of targets of different scales, the model can be adapted to target detection of different scales through a large number of training. However, when the number of samples is small, the training samples of different scales will become insufficient. In the process of small sample learning, due to the large scale difference between the support set and the query set images, it brings some difficulties to the detection task.

In order to solve the influence of scale change on the detection and recognition effect of this method, this part introduces a multi-scale FPN module [20]. The module can extract features at different levels and fuse information at different scales, so as to effectively solve the problem of large differences in scales between support set and query set images in small sample learning, and improve the accuracy and stability of detection.

**Figure 6:**
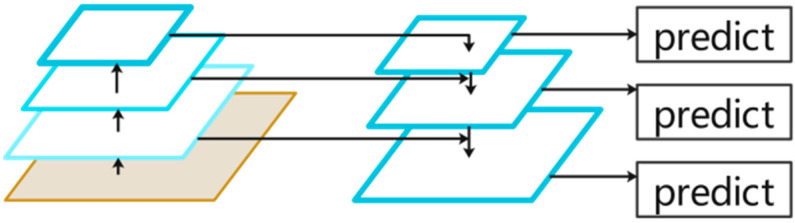
FPN network structure diagram.

FPN is a network module used to solve the problem of object scale change in object detection. The FPN module is mainly composed of a bottom-up feature extraction network, a top-down feature fusion network, and a horizontal connection between them. The FPN module can not only extract features effectively, but also generate feature maps with different resolutions and semantic information according to the scale changes of objects in the image, so as to improve the accuracy of target detection. The FPN structure diagram is shown in Figure 6.

Specifically, FPN uses a bottom-up feature extraction network to extract shallow features in the network. These features have high resolution and rich detail information, but they are gradually lost when the scale becomes larger. At the same time, FPN also uses a top-down feature fusion network to extract deep features in the network. These features have large receptive fields and abstract semantic information, but gradually lose detailed information when the scale becomes small. In order to solve this problem, FPN establishes a horizontal connection between different levels, and fuses features from shallow and deep layers to form a feature pyramid. Each layer in the feature pyramid contains feature information of different scales, so that the network can better adapt to targets of different scales.

## 4. Experiments

### 4.1. Data Preparation

#### 4.1.1. Self-Made Dataset

In this paper, we first use the self-made data set to carry out the preliminary ablation experiment, use the network crawler to capture the traffic sign images in different scenes, and use the LabelImg tool to mark them. LabelImg is a common visual image object annotation software, which is often used in object detection. The label file generated by LabelImg is an XML file, which follows the standard format of PASCAL VOC. The software is open sourced in github.

**Figure 7:**
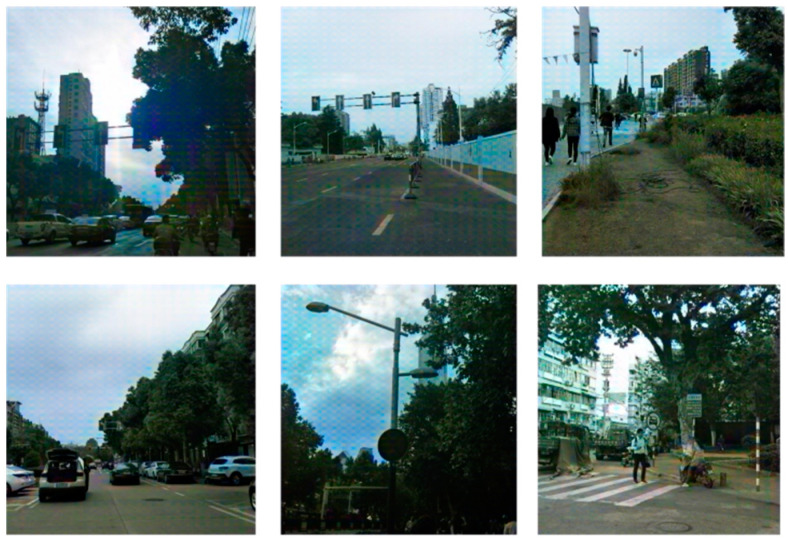
Self-made data set part of the picture.

The self-made dataset contains a total of images of straight, nohook, stop, left, crosswalk, and right. In this part, 15–20 images were randomly selected from these seven categories to form a small sample data set. As shown in Figure 7.

**Figure 8:**
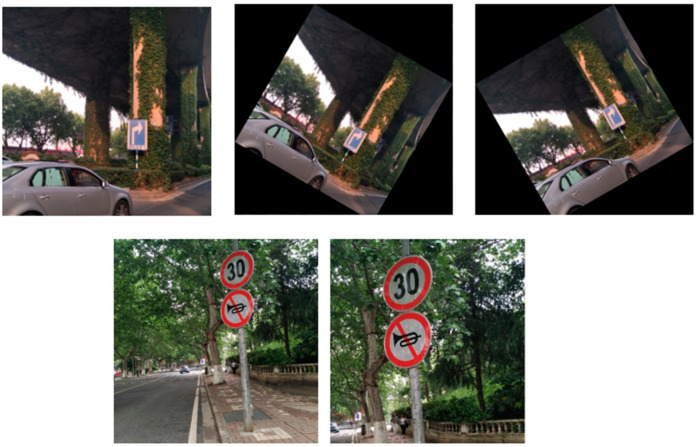
Some pictures of the expanded dataset.

When using self-made data sets for experiments, due to the small number of samples, there will be over-fitting. Therefore, this section first amplifies the data set used, and expands the data set by data augmentation methods such as rotation, clipping, and scaling to balance the data, while improving the robustness of the model and avoiding overfitting. The partially enhanced image is shown in Figure 8.

The number of pairs before and after the enhancement of the dataset is shown in Table 1.

#### 4.1.2. PASCAL VOC Dataset

The PASCAL VOC (VOC) dataset consists of VOC2007 [21] and VOC2012 [22]. There are 20 types of common targets in life (excluding background classes).

For the small sample target detection task, the data set contains three classification criteria: {bird, bus, cow, motorbike, sofa} are selected as the new class in Split1; five categories of {aeroplane, bottle, cow, horse, sofa} are selected as new classes in Split2; and in Split3, {boat, cat, motorbike, sheep, sofa} are selected as new classes.

### 4.2. Evaluation Methods

When using the model for prediction, it is usually necessary to classify the prediction results into positive samples and negative samples. The prediction results can be evaluated by two indicators: precision and recall. The precision rate refers to the proportion of true positive samples in the samples predicted as positive samples, while the recall rate refers to the proportion of true positive samples in the samples predicted as positive samples. The setting of the threshold will affect the calculation results of the precision rate and recall rate, so different combinations of precision rate and recall rate can be obtained by changing the value of the threshold. The precision and recall values under different thresholds are plotted as PR curves. The horizontal axis is the recall rate and the vertical axis is the precision rate. Generally, the PR curve shows an overall trend; that is, the higher the accuracy, the lower the recall rate. The area of the PR curve, also known as average precision (AP), is an important indicator of model performance, as shown in Formula (7).

(7) $$AP=\int_{0}^{1}p\left(r\right)dr$$

AP50 refers to the AP value when the IOU threshold is 0.5; that is, the accuracy of the detection results when the overlap between the detection box and the real box reaches more than 50%. AP75 is the AP value when the overlap between the detection box and the real box reaches more than 75%. These indicators can more comprehensively evaluate the performance of the target detection model and provide more detailed and accurate evaluation results. They play an important role in selecting the appropriate threshold and algorithm optimization.

### 4.3. The Results of Ablation Experiments

#### 4.3.1. RPN Module with Multi-Attention Mechanism

In order to verify the effectiveness of the improved RPN module proposed in this paper, this part designs relevant ablation experiments on self-made data sets. In this section, 5-way 3-shot and 5-way 5-shot tasks are used for testing. The test results are shown in Table 2.

As can be seen from Table 2, after introducing the improved ACBAM module in this paper, in the case of 5-way 3-shot, the average accuracy of the network on AP50 is increased by 1.87%, and the average accuracy on AP75 is increased by 0.93%. In the case of 5-way 5-shot, the average accuracy of the network on AP50 is increased by 2.49%, and the average accuracy on AP75 is increased by 1.07%. According to the above experimental results, the ACBAM attention mechanism proposed in this paper can effectively improve the detection performance of traffic signs in the case of small samples.

#### 4.3.2. Multi-Scale Feature Fusion Module

In order to verify the necessity of introducing multi-scale FPN module, this part designs relevant ablation experiments on self-made data sets. In this section, two groups of tasks, 5-way 3-shot and 5-way 5-shot, are tested. The test results are shown in Table 3.

It can be seen from Table 3 that when the FPN module is introduced at the same time in the query branch and the support branch, the accuracy of the network on AP50 is improved by 2.73%, and the accuracy on AP75 is improved by 0.57% in the case of 5-way 3-shot. In the case of 5-way 5-shot, the accuracy of the network on AP50 is improved by 3.25%, and the average accuracy on AP75 is improved by 1.67%. According to the above experimental results, it can be seen that in the case of small samples, the introduction of FPN module on both support branch and query branch can effectively improve the detection performance of traffic signs.

#### 4.3.3. Ablation Experiments

In order to verify the effectiveness of each improved method, ablation experiments were performed on self-made data sets. Firstly, the corresponding data enhancement operations, including rotation and mirroring, are carried out on the annotated data sets to achieve the purpose of data balancing. In this section, FSOD is taken as the baseline network, and 5-way 3-shot and 5-way 5-shot tasks are used for training. The aforementioned improvement methods are combined to verify the improvement of network performance by different modules. The results are shown in Table 4.

Comparing baseline with models A, B, and ACBAM-FSOD, it can be seen that the performance of the model is improved by improving the RPN module and introducing the FPN network. The improved ACBAM-FSOD, whether in the 5-way 3-shot task or in the 5-way 5-shot task, achieved better results in AP50 and AP75 evaluation indicators.

**Figure 9:**
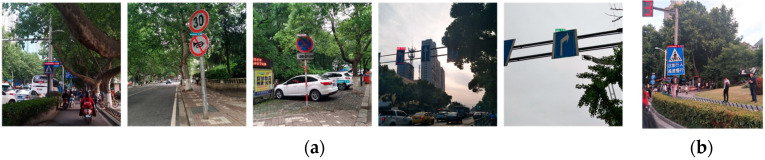
Visualization results of Self-made dataset image detection. Expression for each category in the figure: (**a**) is the detection result of the model on the support set, and (**b**) is the detection result of the model on the query set.

The detection results of the ACBAM-FSOD model on the self-made data set are shown in Figure 9.

### 4.4. The Results of Comparative Experiments

In order to further verify the effectiveness of ACBAM-FSOD, this section conducts comparative experiments on the PASCAL VOC dataset, and selects target detection algorithms such as Meta RCNN [23], TFA (two-stage fine-tuning approach) [24], and MPSR (multi-scale positive sample refinement) [25] for comparison. The algorithms are evaluated according to AP50 (AP value when IOU threshold is 0.5) and AP75 (AP value when IOU threshold is 0.75). In order to make a more comprehensive experimental comparison, this section uses three different division methods, namely, split1, split2, and split3, as shown in 4.1.2. With these three criteria, the model is compared and analyzed experimentally. The experimental results are shown in Table 5.

It can be seen from Table 5 that the detection performance of ACBAM-FSOD on the CCTSDB dataset is partially superior to other algorithms. Compared with the Meta RCNN method, this method has obvious advantages in each task. Compared with the TFA method, when the division criteria are Split1 and Split2, the proposed method has obvious advantages in each task. When the partition condition is Split3, the proposed method has obvious advantages in other tasks except the 5-way 5-shot task. Compared with the MPSR method, the proposed method has obvious advantages in each task when the division standard is Split1. When the splitting criterion is Split2, the proposed method has obvious advantages in other tasks except the 5-way 10-shot task.

**Figure 10:**
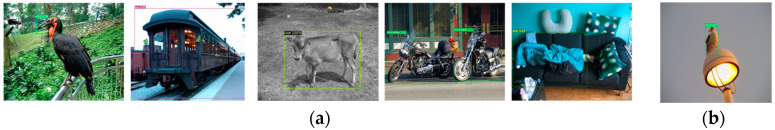
Visualization results of VOC dataset image detection. Expression for each category in the figure: (**a**) is the detection result of the model on the support set, and (**b**) is the detection result of the model on the query set.

The detection results of the ACBAM-FSOD model on the PASCAL VOC dataset are shown in Figure 10.

## 5. Conclusions

The current detection and recognition methods of traffic signs require a large number of data samples for pre-training, the traffic sign update iteration is fast, and the collection and labeling of related data sets will consume a lot of manpower, material, and financial resources. To solve this problem, a series of experimental studies were carried out based on the FSOD model. Aiming at the problems of serious overfitting and excessive candidate boxes unrelated to objects caused by insufficient sample size in the detection and recognition process, this paper proposes adjusting the network structure and a design for an RPN module that integrates improved attention. Aiming at the problem of low detection and recognition accuracy due to the large change of sample scale in the process of detection and recognition, this paper introduces a multi-scale FPN module. Through a series of ablation and comparison experiments, the proposed method shows good performance in small sample target detection tasks.

Because most of the current small sample target detection methods are based on the two-stage target detection method, it is difficult to meet the real-time requirements. At present, most of the research on target detection in the case of small samples pays more attention to the improvement of detection accuracy in different tasks, but less attention to real-time problems. To solve this problem, it is necessary to continue to carry out experimental exploration to further improve the performance of target detection in the case of small samples.

## Figures and Tables

**Table 1:** Number of pairs before and after the enhancement of the dataset.

Category	Number of Data before Enhancement	Number of Data after Enhancement
Straight	15	329
Nohook	18	368
Stop	16	336
Left	18	374
Slow	17	365
Crosswalk	15	312
Right	16	351

**Table 2:** ACBAM-RPN module ablation experiment. Expression for each category in the table: A represents the original channel attention module in the original CBAM, B represents the improved channel attention module, and C represents the spatial attention module in the original CBAM.

Method			Self-Made Dataset (5-Way 3-Shot)		Self-Made Dataset (5-Way 5-Shot)	
A	B	C	AP50 (%)	AP75 (%)	AP50 (%)	AP75 (%)
			14.39	14.39	19.70	15.15
√			14.85	14.39	19.92	15.14
	√		15.34	14.72	21.15	15.28
		√	14.75	14.41	19.83	14.67
√		√	15.11	14.43	20.27	15.71
	√	√	16.26	15.32	22.19	16.22

**Table 3:** FPN module ablation experiment. Expression for each category in the table: A represents the query branch that introduces the FPN module, and B represents the support branch that introduces the FPN module.

Method		Self-Made Dataset (5-Way 3-Shot)		Self-Made Dataset (5-Way 5-Shot)	
A	B	AP50 (%)	AP75 (%)	AP50 (%)	AP75 (%)
		14.39	14.39	19.70	15.15
√		16.19	14.43	21.37	15.72
	√	16.60	14.44	21.21	15.21
√	√	17.12	14.96	22.95	16.82

**Table 4:** Experimental comparisons of each combination in the feature extraction network.

Model	Method		Self-Made Dataset (5-Way 3-Shot)		Self-Made Dataset (5-Way 5-Shot)	
	Improved RPN	FPN	AP50 (%)	AP75 (%)	AP50 (%)	AP 75(%)
Baseline			14.39	14.39	19.70	15.15
A	√		16.26	15.32	21.37	15.72
B		√	17.12	14.96	21.21	15.21
ACBAM-FSOD	√	√	18.66	15.91	22.95	16.82

**Table 5:** Comparative experimental results on public datasets.

Method/Shot	Split1				Split2				Split3			
	1	3	5	10	1	3	5	10	1	3	5	10
Meta RCNN	19.9	35.0	45.7	51.5	10.4	29.6	34.8	45.4	14.3	27.5	41.2	48.1
TFA2	39.8	44.7	55.7	56	23.5	34.1	35.1	39.1	30.8	42.8	49.5	49.8
MPSR 3	41.7	51.4	55.2	61.8	24.4	39.2	39.9	47.8	35.6	42.3	48.0	49.7
Ours	45.8	54.3	56.9	62.1	27.9	40.0	40.4	47.6	34	43.2	48.5	49.8

## Data Availability

Not applicable.
